# Supporting Our Valued Adolescents (SOVA), a Social Media Website for Adolescents with Depression and/or Anxiety: Technological Feasibility, Usability, and Acceptability Study

**DOI:** 10.2196/mental.9441

**Published:** 2018-02-26

**Authors:** Ana Radovic, Theresa Gmelin, Jing Hua, Cassandra Long, Bradley D Stein, Elizabeth Miller

**Affiliations:** ^1^ Department of Pediatrics Children's Hospital of Pittsburgh of UPMC University of Pittsburgh School of Medicine Pittsburgh, PA United States; ^2^ Graduate School of Public Health University of Pittsburgh Pittsburgh, PA United States; ^3^ Research and Development Corporation Pittsburgh, PA United States

**Keywords:** adolescent, adolescent health services, technology, depression, anxiety

## Abstract

**Background:**

Supporting Our Valued Adolescents (SOVA), a social media website for adolescents, was designed to increase mental health literacy and address negative health beliefs toward depression and/or anxiety diagnosis and treatment. This stakeholder-informed site underwent iterative user testing to evolve into its current version with daily blog posts, round-the-clock site moderation, and Web-based peer interaction to create an online support community.

**Objective:**

The aim of this study was to evaluate the technological feasibility (at least 100 users on the site, logging in 12 to 18 times in the first 6 weeks) and acceptability of the SOVA site determined by the System Usability Scale (SUS).

**Methods:**

Adolescents and young adults (aged 14-26 years) with a self-reported history of depressive and/or anxiety symptoms were recruited to access the research website (sova.pitt.edu). Participants were screened out if they reported active suicidality or a prior suicide attempt. Baseline survey measures included demographics, symptomatology using the Patient Health Questionnaire-9 modified for adolescents (PHQ-9A) and Screen for Child Anxiety Related Disorders (SCARED-C), and mental health treatment history. The 6-week follow-up measures taken in addition to the symptomatology, included feasibility (total number of log-ins), usability, and acceptability of SOVA using SUS.

**Results:**

Most of the 96 participants identified as female (75% [72/96]) and white (67% [64/96]). Most participants (73% [70/96]) reported having taken prior professional psychological help. The average PHQ-9A score was 11.8 (SD 5.5), and for SCARED-C, 85% (80/94) of the participants reported a score consistent with being susceptible to a diagnosed anxiety disorder. There were 46% (41/90) of eligible users who ever logged in. Out of the total users who ever logged in, the mean of total log-ins over the entire study was 4.1 (SD 6.9). Median number of users rated the user-friendliness of the site as “good.” The average SUS score was 71.2% (SD 18.7), or a “C-grade,” which correlated to an acceptable range. The participants reported to have liked the “easy-to-understand format” and “positive, helpful atmosphere,” but they also reported a desire for greater social interaction. Iterative recruitment resulted in incremental improvements to the site.

**Conclusions:**

The SOVA site met feasibility goals of recruiting almost 100 users and establishing acceptable usability. Subsequent interventions are planned to increase site engagement and to evaluate efficacy in increasing uptake of primary care–recommended depression and/or anxiety treatment.

## Introduction

### Background

Suicide is the second most common cause of death in adolescents and young adults in the United States [1]; the most common risk factor for suicide is mental illness [2,3]. Alarmingly, as per a 2017 national survey, less than one third of suicidal youth had used mental health services [4]. A primary predictor of not utilizing mental health services is the harboring of negative beliefs about mental illness and treatment seeking [5]. For example, youth who do not seek help have a higher degree of self-stigma, as well as self-reliance, or a belief that they do not need others’ help [6]. Digital health interventions may be a promising avenue to change these negative health beliefs, particularly among young people. Digital interventions can reach a wide audience [7] of young people who commonly use the Internet to find health resources [8] and specifically talk about their mental health [9,10], thereby serving as a potential bridge to face-to-face psychotherapy [11,12].

To seize this potential, we designed a social media website for adolescents called SOVA (Supporting Our Valued Adolescents) and a separate companion website for parents called wiseSOVA (not discussed in this paper). SOVA aims to (1) challenge negative health beliefs and increase depression and anxiety knowledge through daily blog posts enhanced with peer commentary, (2) promote social support through Web-based peer interactions, and (3) encourage parent-adolescent mental health offline communication through same-day blog posts with questions for discussion. SOVA’s goal is to increase the perceived need for services in both, adolescents referred for treatment and their parents, ultimately leading to increasing the use of adolescent mental health services.

### Objectives

From the inception of the SOVA sites, we knew we would need to use multiple strategies to buffer against the lack of engagement which affects many ehealth interventions [13]. Our main strategy was to involve end users [14] to help increase engagement [15] by recognizing important concerns in the specific population. For example, for adolescents and young adults with mental health problems, a salient concern is ensuring confidentiality [16]. To accomplish this, we took a stepwise approach, incorporating technology development principles and behavioral intervention testing. This approach is based on a slightly modified structure recommended by the Office of Behavioral and Social Sciences Research and multiple NIH institution collaboration for behavioral intervention development, the ORBIT model [17]. The phase 1 design stage involved formative work and iterative user-testing with adolescent or young adults, parents, and provider stakeholders. This led to the development of the current versions of the sites with daily blog posts, round-the-clock site moderation by behavioral health professionals or trainees, and Web-based interaction with peers interacting in an online support community (one for adolescents and young adults and another one for parents) [18]. As part of phase 2 of the ORBIT model, we conducted a preparatory study to refine the SOVA intervention and determine its usability through beta testing. Specifically, we sought to evaluate the technological feasibility, defined as the ability to recruit users to the site, and frequency of log-ins, as well as the acceptability of the SOVA site, as determined by the System Usability Scale (SUS) [19]. We expected to recruit 100 adolescents and young adults to the site and that they would log in for a mean of 12 to 18 times over the 6-week study. We expected that users would find SOVA to have at least “good” user-friendliness and a corresponding SUS acceptability mean of 73 [19]. This manuscript provides usability data only on SOVA, the adolescent or young adult site, and describes preliminary changes in some potential outcomes of interest, including depression and anxiety symptoms and positive youth development characteristics, such as caring and connection.

## Methods

### Intervention

**Figure 1:**
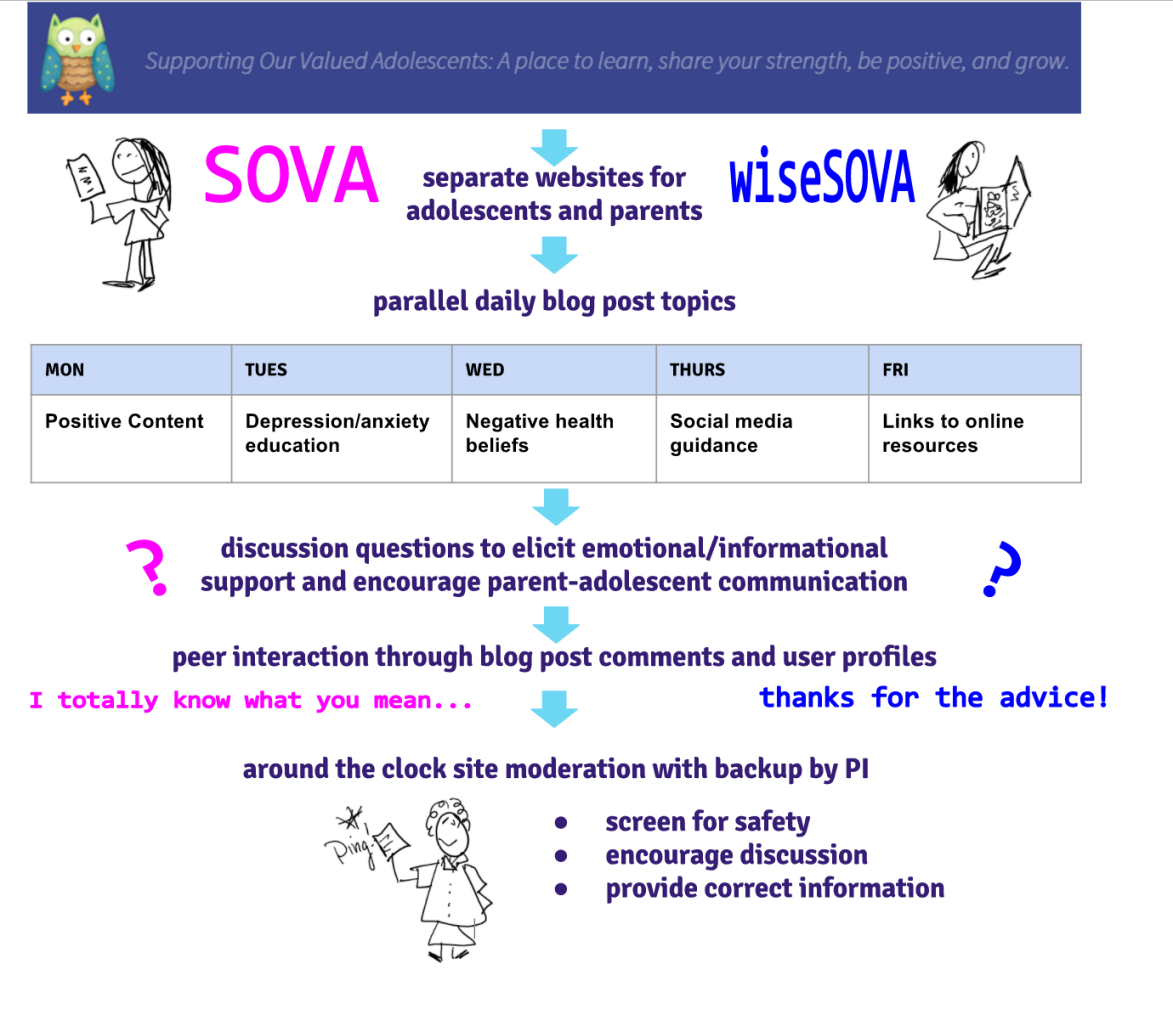
Supporting Our Valued Adolescents (SOVA) key intervention components.

The SOVA site's process of iterative development and design is described in detail elsewhere [18]. A secure password-protected site was developed (sova.pitt.edu) using WordPress [20], a popular content-management system frequently updated for function and security [21]. A simple central interface allows a novice to add and organize site content [22] but results in a professional-looking website in a blog post format [20,21]. The research team contributed to daily weekday blog posts written with a youth audience in mind, tailored for cultural sensitivity and reviewed for health literacy [23] and readability [24] of grade level 8 or lower. Topics were categorized under the headings (1) “Be Positive” or posts with positive content, such as an inspiring quote or video (eg, “The Power of Hugs”); (2) “Educate Yourself” or posts addressing depression and anxiety psychoeducation [25] or negative health beliefs that may prevent someone from seeking care [26-29] (eg, “Because I felt like I didn’t deserve to get better”); (3) “Social Media Guide” or posts providing information or guidance on social media use based on our prior qualitative study with depressed youth [30] (eg, “Instagram’s newest safety tool”); and (4) “Links” or posts describing an existing resource (eg, “Getting Help: The National Suicide Prevention Hotline”; see Figure 1). At the bottom of each post, a question was placed to promote user discussion (eg, “Have you ever felt guilty for something you realized later was not actually your fault?”). A graduate student of information science addressed technical challenges, created a user-tracking mechanism and data-visualization module. Moderators for the website were behavioral health graduate students (ie, social work and psychology) and clinician members of the research team. Each moderator participated in an in-person training session and received a detailed protocol. Weekly group supervision sessions were held with the principal investigator and a licensed social worker research assistant.

### Study Participants and Setting

Adolescents and young adults (AYA) aged 14 to 26 years were recruited in-person from clinical settings by behavioral health clinicians and from online websites (eg, Craigslist, University of Pittsburgh Research Registry). Young adults up to the age of 26 years were included as the online community we sought to form that could benefit from peer involvement from young adults who had had positive experiences in the mental health care system and exhibited resilience [31]. Recruitment advertising was directed toward those AYA who were experiencing symptoms of depression and/or anxiety and were interested in providing feedback about a new website that would allow anonymous Web-based interaction with other people their age who were also experiencing these symptoms. Interested participants were asked to go to the website (sova.pitt.edu) and were required to register and log in to access any site content. Toward the end of the study, after 80% (77/96) of the sample had been recruited, we changed this log-in requirement. This was based on participant feedback that engagement may increase if we made some website content public so that participants could view it before deciding whether to log in and enroll in the study. After this change, the content of all blog articles was made public, but online community features (eg, creating a profile, creating and viewing comments on blog posts, and receiving email updates on new blog posts) still required the users to log in.

### Data Collection

After creating a username, password, and agreeing with a set of common-ground rules emphasizing anonymity on the main website, participants were automatically redirected to a Web-based survey (Qualtrics, Provo, UT). Individuals aged 14 to 26 years were included if they self-reported a history of depression and/or anxiety symptoms, had Internet and email access, could read and write in English, and had completed the 6th grade. We obtained a waiver of parental permission because of anticipated difficulty with recruitment due to the study being online, and because minors aged 14 years and older can seek mental health services without parental permission in Pennsylvania, United States. Due to the unknown safety profile of the intervention, we excluded participants with active suicidality, defined by thoughts with intent to act on these thoughts, or a history of a suicide attempt. Participants screening in were asked for their contact information and for 1 supportive adult; this was described as a requirement of the study for safety reasons. Those screening in would be redirected to complete a baseline 52-item Web-based survey. After 6 weeks, they received a follow-up 63-item Web-based survey by email. Passive data were also collected regarding the number of log-ins and text from comments in response to blog posts. Participants received compensation in the form of a prepaid debit card on the completion of the first and 6-week surveys.

### Measures

#### Demographics

At baseline, participants were asked their age, gender, and race.

#### Depression and Anxiety

Depression symptoms were measured using the Patient Health Questionnaire-9 Item (PHQ-9) modified for adolescent use and using the cut-off score of 11 for detecting major depression [32]. Higher PHQ-9 scores have been correlated with greater functional impairment and parental report of psychosocial problems.

Anxiety symptoms were measured using the 5-item version of the Screen for Child Anxiety Related Emotional Disorders (5-item SCARED-C) [33]. This brief version of SCARED-C, including questions regarding panic or somatic, general anxiety, separation anxiety, social phobia, and school phobia has been found to have 74% sensitivity and 73% specificity for detecting clinically significant anxiety using a score of 3 or greater.

Mental health treatment history was ascertained by asking AYA whether they had ever received treatment from a professional psychologist or counselor and/or taken a medication such as an antidepressant [34].

#### Positive Youth Development Scale

The Positive Youth Development 17-item Very Short Form (PYD-VSF) is an abbreviated version of the full PYD, which has been used to measure positive attributes in AYA based on the Lerner and Lerner Five Cs Model of PYD [35]. This model operationalizes PYD by assessing (possible subscale score values in parentheses) competence (1-12), confidence (1-13), character (1-19), connection (1-20), and caring (1-15), with the total score ranging from 1 to 79. PYD-VSF has been validated in multiple groups of adolescents [36].

#### Feasibility: Frequency and Patterns of Use

User log-in data over the initial 6 weeks of site use and afterwards (some individuals continued to use the site after 6 weeks) was collected, as well as the frequency of viewing specific blog post categories, such as Education (twice as many posts as other categories), Social Media, Positivity, and Resources was also reported. Aggregate data of site use was also collected by views of unique Internet protocol (IP) addresses, filtering out the IP addresses of the study team. A data visualization module was created to view daily, weekly, and monthly log-ins, unique IP addresses, and blog post article comments on the same display over time and allowed notation of events that may affect use (eg, opening site articles to public).

#### Usability and Acceptability

Website usability and acceptability were measured using the modified SUS [19] and 2 open-ended questions. The modified SUS consists of 10 questions regarding usability with responses on a 1 to 5 Likert scale in terms of agreement and an additional 11th question with response on an adjective rating scale. Scores based on the first 10 questions were manipulated to a 0 to 4 rating and multiplied by 2.5 to get to a score within a range of 0 to 100, with higher scores denoting higher usability. Question 11 asks the user to rate the overall user-friendliness of the site as worst imaginable, awful, poor, OK, good, excellent, or best imaginable. Bangor et al reviewed 200 studies and found the highest quarter of study means for SUS ranged from 78.51 to 93.93 [19]. Factor analysis has shown only 1 significant factor for the 10 SUS statements, implying a good fit for usability, and reliability analysis has shown a Cronbach alpha of .911 [19]. The following two open-ended questions were asked: (1) “What did you like about this website?” and (2) “What would you change about the website?” with free-text entry responses. Phone or email interviews were also offered to participants to provide further feedback, but only 4 individuals chose to participate.

### Analysis

Descriptive analyses were used for summary statistics of all measures listed above. The primary outcome for feasibility was mean number of log-ins over a 6-week period, and for usability and acceptability, the SUS mean score. Paired *t* tests (for continuous outcomes) and McNemar tests (for categorical outcomes) were used to compare change in pre- and postintervention repeated measures in the same sample which completed both baseline and 6-week measures. Before the study, we calculated that a sample size of 100 AYA would give sufficient precision to estimate the mean number of log-ins and mean usability score within 0.20 standard deviations (with the 95% CI). SPSS Version 24.0 (IBM) was used for statistical analyses. Open-ended responses and interviews were reviewed by 2 team members using thematic coding to gather specific content about usability. This study was approved by the University of Pittsburgh Human Research Protection Office.

## Results

**Figure 2:**
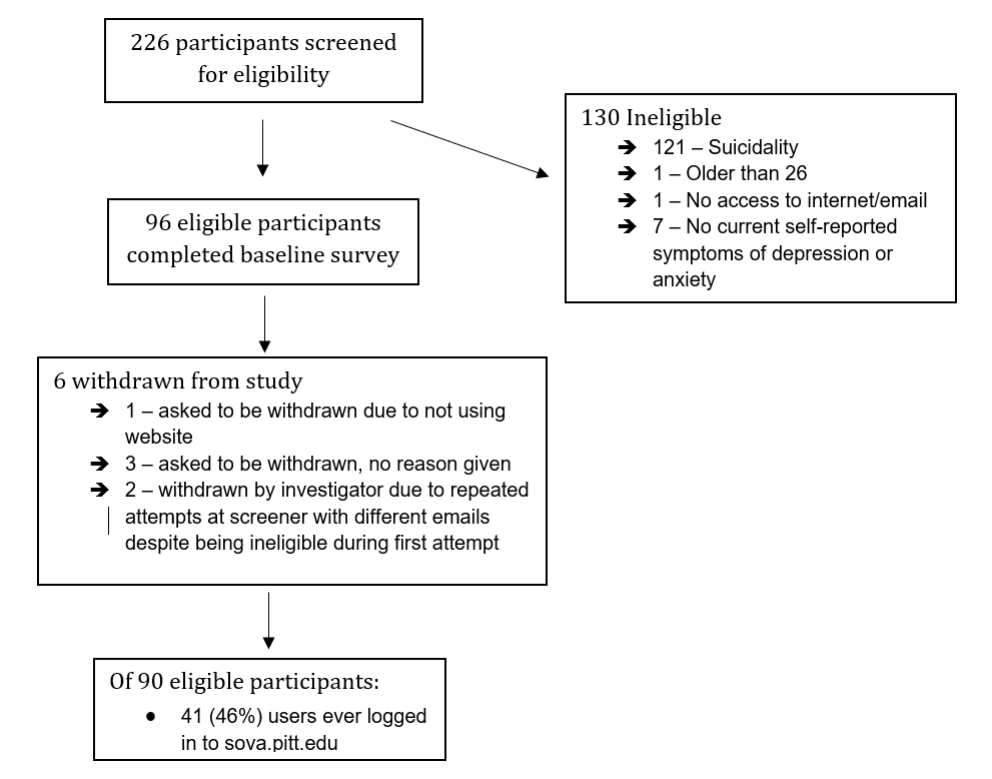
Participant flowchart.

Screening for eligibility took place with 226 individuals, of whom 130 were ineligible, mostly because of suicidality (N=121; see Figure 2). Out of 96 eligible participants, all completed the baseline survey, but 6 withdrew or were withdrawn afterwards by the research team (eg, due to attempts to complete the survey multiple times) and did not create a username for the site. Out of these 90 users, 70 completed the 6-week survey (78% [70/90]), although the N reported for some measures below is lower due to incomplete completion of specific survey items.

The baseline sample (Table 1) included 15 adolescents (aged 14-19 years) and 80 young adults (aged 20-26 years; 1 missing age data). Race was fairly representative of US demographics, except for twice as high a rate of Asian or Pacific Islander. At baseline, the mean PHQ-9 score was 11.8 (SD 5.5), which is above the cut-off score for depression in adolescents [32], and about 30% reported moderately severe to severe symptoms. Using SCARED-C, 85% (80/94) of the participants reported a score consistent with being susceptible to being diagnosed with an anxiety disorder. Over half of the participants had ever received a medication such as an antidepressant (57% [55/96]) or help from a professional psychologist or counselor (73% [70/96]). The baseline PYD-VSF total score was 54.4 (SD 7.5), with the subscale scores listed in Table 1.

### Feasibility

There were 46% (41/90) of participants who ever logged in. As 61 participants completed the 6-week survey on usability, as many as 13 may have viewed content without logging in once it was public or only viewed the content in their notification email, but this cannot be determined from the data collected because of anonymity of 6-week data collection and inability to match it to usernames. Out of those who ever logged in (users), the mean total log-ins over the initial 6 weeks were 1.9 (SD 2.3) and over the total study were 4.1 (SD 6.9; see Table 2). The most frequently viewed blog posts over the initial 6 weeks were education posts (of which there were double the number of posts) with a mean of 5.1 (SD 14.7) views, and the second most-commonly viewed posts were positivity posts with a mean of 2.8 (SD 6.9).

**Figure 3:**
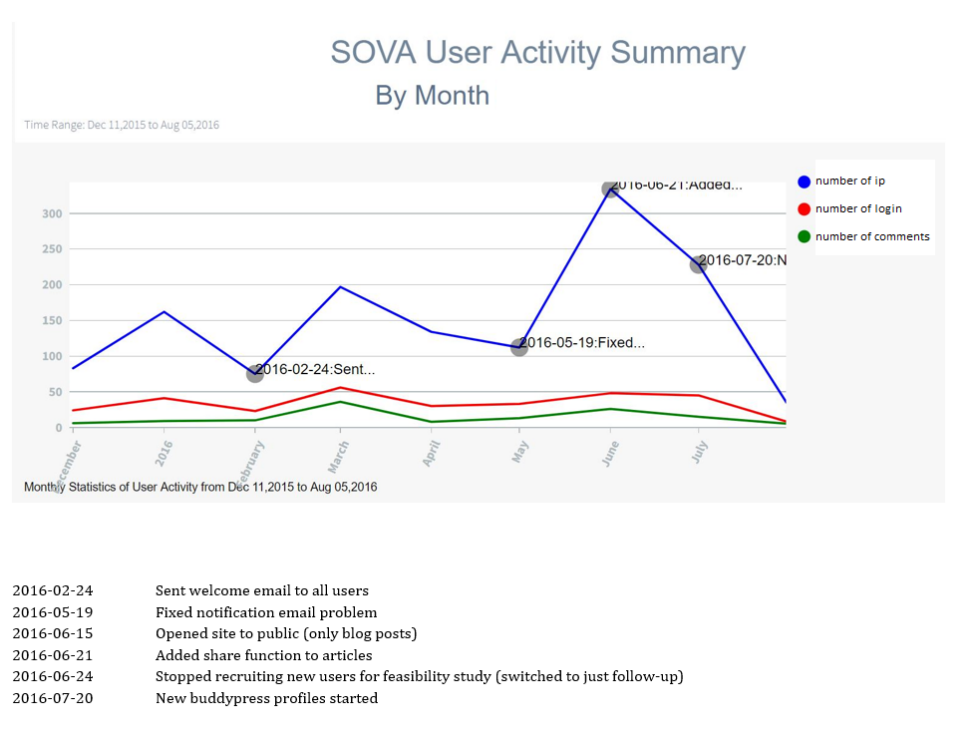
Data visualization.

Data visualization showed a sharp increase in site views by unique IP addresses in June 2016 after the site blog posts were made public and a problem with users not getting notification emails was resolved (Figure 3). Beside this problem, there were no major site errors and no safety concerns. Figure 3 shows in red, the number of unique views of the site per month (omitting the study team’s views); in blue, the number of log-ins per month ranged 21 to 56 (until October when the study was almost over); and in green, the number of comments per month ranged 8 to 36.

**Table 1:** Demographics and baseline measures of study population (N=96).

Variable		Value
Age in years, median (range)		23 (14-26)
**Gender, n (%)**		
	Female	72 (75)
	Female to male transgender	0 (0)
	Male	21 (22)
	Male to female transgender	0 (0)
	Not sure	0 (0)
	Other	3 (3)
**Race, n (%)^a^**		
	White	64 (67)
	Black	14 (15)
	Asian/Pacific Islander	12 (12)
	Hispanic	7 (7)
	North American Native	1 (1)
	Other	0 (0)
	Don’t want to answer	2 (2)
Depressive symptoms: PHQ-9 Score^b^, mean (SD)^c^		11.8 (5.5)
Feeling sad most days in the past year, n (%)		67 (70)
**Difficulty experienced with normal functioning, n (%)^c^**		
	Not difficult at all	9 (9)
	Somewhat difficult	55 (58)
	Very difficult	23 (24)
	Extremely difficult	8 (8)
**Depression severity (PHQ-9 Score), n (%)^c^**		
	None (1-4)	8 (8)
	Mild (5-9)	30 (32)
	Moderate (10-14)	27 (28)
	Moderately severe (15-19)	21 (22)
	Severe (20-27)	9 (9)
SCARED-C^d^ score consistent with anxiety (≥3), n (%)^e^		80 (85)
**Treatment history (yes to having ever received), n (%)**		
	Professional psychologist or counselor	70 (73)
	Medication like antidepressants	55 (57)
**Positive Youth Development-VSF score, median (SD)^f^**		54.4 (7.5)
	Competence	7.6 (2.0)
	Confidence	8.0 (2.5)
	Character	13.6 (2.6)
	Caring	13.0 (2.2)
	Connection	12.2 (3.2)

^a^Percentage may equal greater than 100 due to participants answering more than one racial category.

^b^PHQ-9: Patient Health Questionnaire-9 modified for adolescents.

^c^N=95 due to exclusion of those who did not answer all PHQ-9 questions.

^d^SCARED-C: 5-item Screen for Child Anxiety Related Emotional Disorders.

^e^N=94 due to those who did not answer all SCARED-C questions.

^f^N=92 due to those who did not answer questions for all PYD categories.

**Table 2:** Feasibility and usability of Supporting Our Valued Adolescents (SOVA).

Outcomes				Value
**Frequency of use, mean (SD)**				
	**Total log-ins/user over initial 6 weeks of use^a^**			
		All users		0.9 (1.8)
		Users who ever logged in		1.9 (2.3)
	**Total log-ins/user ever**			
		All users		1.8 (5.0)
		Users who ever logged in		4.1 (6.9)
**Patterns of use over initial 6 weeks^b^, mean (SD)**				
	**Total education blog post views/user**			
		All users		2.3 (10.1)
		Users who ever logged in		5.1 (14.7)
	**Total social media blog post views/user**			
		All users		0.9 (3.7)
		Users who ever logged in		2.0 (5.3)
	**Total positivity blog post views/user**			
		All users		1.2 (4.8)
		Users who ever logged in		2.8 (6.9)
	**Total resources blog post views/user**			
		All users		0.8 (3.4)
		Users who ever logged in		1.8 (4.9)
System Usability Scale score^c^, mean (SD)				71.2 (18.7)
**User-friendliness of site, n (%)**				
	Worst imaginable			0 (0)
	Awful			1 (2)
	Poor			3 (5)
	OK			11 (18)
	Good			19 (31)
	Excellent			21 (34)
	Best imaginable			6 (10)

^a^Data available for 90 accounts as 6 users requested to be withdrawn.

^b^Differences were not statistically significant (*P*>.05) using ANOVA.

^c^N=61 due to loss to follow-up; 70 users completed follow-up but not all completed each measure.

### Usability and Acceptability

The median number of users thought the user-friendliness of the site was “good.” Over a third of users (34% [21/61]) thought the user-friendliness of the site was excellent (Table 2). The average SUS scale score was 71.2 (SD 18.7) or a C-grade, which is within an acceptable range [19]. From these, 49 users responded to the open-ended question “What did you like about the website?.” They liked the “easy-to-understand format” and “positive, helpful atmosphere,” but desired greater social interaction. They liked that (1) “new content was added,”(2) “spread out to make me see every topic,” (3) “very interactive,” and (4) “I liked how one person's comment would trigger a larger conversation.”

Only 4 users participated in poststudy interviews. One interviewee commented the site was, “better than I expected it to be” and later clarified:

I didn’t expect [the site] to be that very in-depth. I thought it was very straightforward, but it was very in-depth and very fun to be on. The blog posts on positive quotes and stories, I’d say they felt very uplifting actually. I really enjoyed it. It felt like I wasn’t reading something in a psychological book. It felt like on a personal level.

ID 3

**Figure 4:**
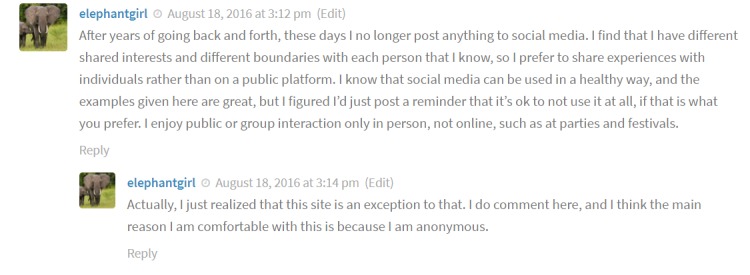
User commenting on anonymity.

On comments to an article on what to share and what not to share on social media, users remarked that although they don’t feel comfortable posting on other websites, they do feel comfortable on sova.pitt.edu due to its anonymity (Figure 4).

In open-ended questions, remarks on what to change mostly centered around increasing interactivity of the site and including less structured ways for users to communicate, such as on a discussion forum. In poststudy interviews, users remarked an app would improve usability due to logging in:

Definitely now with smartphones like if there’s an app it’s so much better than having to log onto the Internet.

ID 2

When asked about a potential future direction of including peer users who compose their own blog posts, a user remarked:

I think that’s a great idea. I think that’s what makes it more likeable you know when people have their own input so they can share their own stories. They write their own journals per se about their experiences. That’s a good idea.

ID 3

### Changes in Mental Health and Social Outcomes

Due to loss to follow-up and missing data, only 57 participants completed enough of the 6-week survey to calculate scoring scales for depression, anxiety, and positive youth development.

There was a statistically significant *(P*=.04) decrease in depression symptoms from baseline to 6 weeks, although the change in score (1.4) was less clinically significant. There was no change in the number of AYA with a SCARED-C score consistent with anxiety (*P*=.34) There was not a significant increase in the number of AYA accessing mental health treatment at follow-up, although the population purposely recruited for this study already had higher than average levels of accessing treatment (Table 3).

Overall, there was a statistically significant positive change in mean total PYD-SF from baseline (54.0, SD 7.6 ) to 6-week (57.6, SD 8.0), *t*_56_=4.32, *P*<.001, with significant increases in subscale scores for competence, confidence, and connection and nonsignificant increases in character and caring (Table 4).

**Figure 5:**
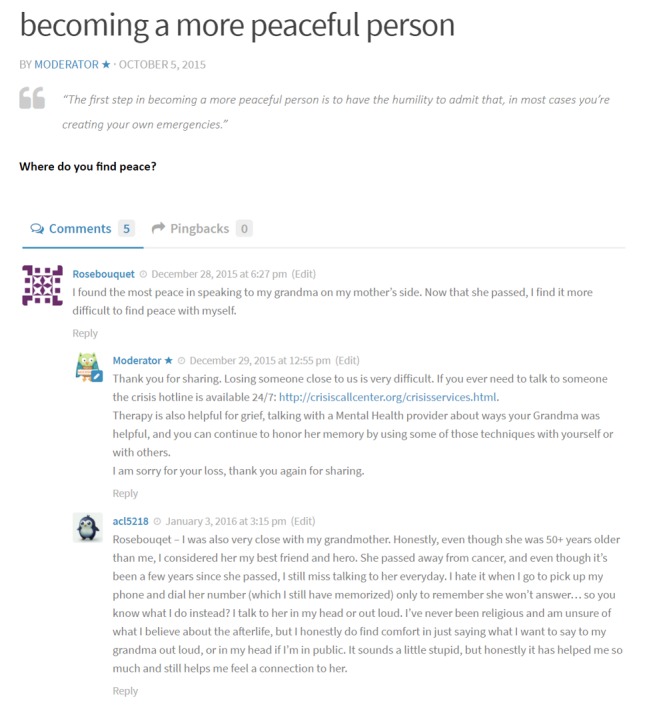
User commenting on providing support.

Participants commenting on blog posts shared personal stories and support (Figure 5).

**Table 3:** Change in depression, anxious symptoms and obtaining treatment at follow-up, N=56. N differs from original group due to loss to follow-up and incomplete data, for example, not completing full scale.

Outcome		Baseline	6-week	Test parameter	Difference	*P* value
PHQ-9^a^ score, mean (SD)		11.6 (5.1)	10.2 (6.1)	Paired *t* test	*t* (df)=2.066 (55)	*P*=.04
SCARED-C^b^ score, score consistent with anxiety (≥3), n (%)		48 (86)	49 (88)	McNemar test	N=56	*P*=.34
**Treatment history (yes to having ever received), n (%)**				
	Professional psychologist/counselor	43 (66)	45 (69)	McNemar test	N=57	*P*=.34
	Medication such as antidepressant	32 (56)	34 (52)	McNemar test	N=57	*P*>.99

^a^PHQ-9: Patient Health Questionnaire-9 modified for adolescents.

^b^SCARED-C: 5-item Screen for Child Anxiety Related Emotional Disorders.

**Table 4:** Changes in Positive Youth Development-Short Form score (N=57). N differs from original group due to loss to follow-up.

Outcome		Baseline	6-week	Test parameter	Difference	*P* value
**PYD-SF^a^score, mean (SD)**		54.0 (7.6)	57.6 (8.0)	Paired *t* test	*t* (df)=−4.32 (56)	*P*<.001
	Competence	7.6 (1.9)	8.2 (2.1)	Paired *t* test	*t* (df)=−2.87 (57)	*P*=.006
	Confidence	7.9 (2.5)	8.6 (2.3)	Paired *t* test	*t* (df)=−2.27 (57)	*P*=.03
	Character	13.4 (2.6)	14.0 (3.1)	Paired *t* test	*t* (df)=−1.11 (57)	*P*=.27
	Caring	12.9 (2.2)	13.2 (2.2)	Paired *t* test	*t* (df)=−0.48 (56)	*P*=.63
	Connection	12.2 (3.2)	13.6 (3.1)	Paired *t* test	*t* (df)=−3.48 (56)	*P*=.001

^a^PYD-SF: Positive Youth Development-Short Form.

## Discussion

### Principal Findings

The aim of this study was to determine the usability of a social media website designed to challenge negative health beliefs and increase depression and anxiety knowledge in adolescents and young adults through daily blog posts enhanced with peer commentary from an online community. We found that maintaining the site was technologically feasible as we experienced very few major errors, aside from finding that notification emails were not being sent because of incorrect settings. We were able to moderate the site and examine all new content in a timely manner. Additionally, there were no safety concerns identified. Feasibility goals were not fully achieved. While we were able to recruit about 100 AYA to the study, which implied interest about the site, only about half of the users ever logged in. We expected that all users would log in and the mean number of log-ins to the site would be 12 to 18 over the 6-week study, but the actual mean of 2 log-ins over the first 6 weeks was much lower than the mean value we expected. We reached our usability goals and found that the median number of users found that SOVA sites had “good” user-friendliness and the SUS scale acceptability mean was 71.2, only slightly lower than our goal of 73. We also found users in this study experienced a slight decrease in depression symptoms and increase in competence, confidence, and connection.

We think we were able to achieve good usability results mainly because of the stepwise stakeholder-informed approach we took, which employed human computer interaction techniques and uncovered user preferences such as a desire for anonymity and functionality to allow them to share their experiences with others [18]. Like other ehealth interventions, we struggled to achieve adequate engagement defined as users logging in and commenting on blog posts, likely due to not having directed engagement interventions in this part of the design and refinement process. One intervention which did seem to increase views, especially from the public, included removing the requirement to log-in to read blog posts and only requiring log-in to join the online community (Figure 3). Initially, due to the at-risk nature of the studied population, we refrained from doing this until we had several months of recruitment without any safety concerns. This is an example of how throughout the design process of this intervention, we had to make multiple decisions that balanced the goal of increasing usability while meeting the goals of the research intervention in this specific population, including safety. Another example is not permitting private conversations between individual users or posts on a newsfeed. Although those features were available in the WordPress plug-in used to employ social media features, Buddy Press [37], these would be too difficult and time-consuming for our team to moderate. Initially, we did try discussion boards, which were easier to moderate, but then removed them in an effort to concentrate the growing online community in fewer locations (only within replies to blog posts) and to avoid the “empty-room phenomenon” (ie, when 1 user comments, there are a low number of concurrent users available to respond) [38].

A review of the literature finds few comparable studies in adolescent and young adult mental health which were tested for usability in the same stage of development or having similar intervention components or goals. The most comparable is a recent study evaluating the feasibility and acceptability of a minimal viability version of ProjectTECH, a Web-based skill-building intervention for adolescents at risk for depression including peer support, in 4 groups of 8 to 12 individuals over an 8-week period [39]. ProjectTECH found a higher median number of log-ins of 26 over an 8-week trial although the log-ins decreased over time and had a lower mean SUS scale score of 67.5 (SD 18.1). The log-in number in this study may have been higher due to the user being aware of planned lessons and a desire to move through materials, as well as phone contact with the research team at recruitment and follow-up, and the use of peer moderators. Compared with this, SOVA did not provide users with instructions on how to use the site and direct communication (beside templated emails) occurred only if necessary, thus limiting opportunities for rapport-building with participants. Also, our moderators are team members. Through further adolescent and young adult stakeholder feedback, we have now incorporated a “how to use the site” video to the current version of SOVA and the involvement of peer bloggers who also frequently comment. In recruitment for future studies, we also plan to use an introductory phone call as an on-boarding process for using the site and to answer participant questions before use.

Web-based interventions to facilitate mental health help-seeking in young adults are feasible [40] and can increase readiness to seek help and decrease stigma [41] and can be used to increase mental health literacy [42,43]. Although Web-based interventions have had positive effects, problems with lack of user engagement with them, especially in less controlled trials and settings, are well-described [44-46]. Reasons for lower than anticipated engagement with our intervention include (1) newness of the site and 100 users recruited slowly over time so that at any moment, an “empty room” may be experienced [38]; (2) high dropout rates experienced in most ehealth trials or “law of attrition,” where many initial users quickly stop accessing the site [13]; and (3) “superuser” effect or 1% rule, where 90% users observe, 9% users contribute sparingly, and 1% users contribute most new content; demonstrated across even long-standing digital health social networks [45]. SOVA users actually outperformed the “90-9-1” rule, as out of the initial almost 100 users, 16 users ever commented and 5 users commented more than 5 times. Unanswered questions remain for technology interventions regarding what amount of engagement is sufficient to achieve the desired health outcome and which techniques increase user engagement [14].

A recent review of 19 Internet-based cognitive behavioral therapy programs for adolescent depression found that some techniques may increase user engagement. For example, real-time guidance, surface credibility or a competent “look and feel” of the site, including video, animation, and interactive exercises, tailoring, and self-monitoring components [47]. Other techniques, including employing peer support [48], incorporating gamification [49], using a supportive accountability model [50], and using mobile phone apps may help increase adherence to adolescent Web-based interventions [39]. A Web-based social media intervention for depression relapse prevention called “Rebound,” which employed both supportive accountability and peer moderators, was found to be acceptable, feasible, highly usable, and safe; and young people with major depression also experienced improvement in their depression scores with this [51].

This study was not designed to test effectiveness, but the direction of slightly decreasing depression symptoms is encouraging. Overall, the AYA participating in this study had high levels of caring and altruistic intentions. Contributing to Web-based interventions which have a goal of sharing experiences in a safe and positive environment may offer opportunities for these AYA to increase aspects of positive development, especially competence, confidence, and connection. Recruiting new users to the site who have high levels of the caring characteristic and have a desire to share what they have learned about being mentally healthy may be a method to increase site engagement as well.

### Limitations

There are several limitations of this study. Due to finding that anonymity was important to users in our previous design study [18], we used a respondent-driven personal identifier code [52] to preserve confidentiality in survey data collection. This in turn limited our ability to confirm whether the 6-week survey respondents included users who had in fact viewed site content. Due to recruitment being online and the majority recruited through Craiglist, there was a high loss to follow-up, and a relatively higher number of young adults were recruited. Although this limits generalizability, the young adults may have been also able to reflect on their experiences throughout adolescence when providing feedback. More than half of the group screened out due to suicidality and mostly due to a history of a previous suicide attempt; usability results may have differed in this group. Due to the unknown safety profile of the intervention before beta testing, we directed recruitment toward a sample which had experienced depression and anxiety but had mostly received treatment. As we found no safety risk in this study, in future studies we will also include those participants who have had a history of suicide attempt but have received treatment. There was no comparison group for the exploratory outcomes, although this was not needed in the overall aim of this study, that is, feasibility and usability. Due to the iterative nature of Internet interventions and differential length of time that different users would participate on the site, the intervention exposure is likely different for each user, albeit this did not detract us from our goal to achieve a desirable usability score. Iterative recruitment resulted in incremental improvements to the site, including opening the blog part of the site to nonusers and correcting a problem with sending daily emails, as well as upgrading to a better user profile design and comment notification. Although there is a risk in a randomized trial to intervention cross-over and limitations to capturing data, we learned that allowing potential participants to view part of a Web-based intervention and “test drive” it (ie, viewing blog articles) before full use and study participation (ie, logging in) simulates more of a real-world experience of trying out a technology intervention before subscribing to it, which may improve user engagement.

### Conclusions

In conclusion, we found that using a stakeholder-informed user design process [18] may increase the subsequent usability of Web-based interventions directed at adolescents and young adults with depression and/or anxiety. Additionally, including adolescents and young adults in shaping Web-based health interventions may take advantage of their preexisting altruism and desire to help peers and help them to develop their strengths. In anticipation of difficulties with engagement, specific procedures need to be incorporated as part of the design process. Future engagement interventions for SOVA will include app development, use of peer bloggers, gamification, and incorporating the supportive accountability model in moderator interactions [50].

## Abbreviations

AYA: Adolescents and young adults
IP: Internet protocol
PHQ-9: Patient Health Questionnaire-9
PHQ-9A: Patient Health Questionnaire-9 modified for adolescents
PYD-VSF: Positive Youth Development-Very Short Form
SCARED: Screen for Child Anxiety Related Emotional Disorders, 5-item version
SOVA: Supporting Our Valued Adolescents
SUS: System Usability Scale
